# HZ08 Reverse P-Glycoprotein Mediated Multidrug Resistance *In Vitro* and *In Vivo*

**DOI:** 10.1371/journal.pone.0116886

**Published:** 2015-02-17

**Authors:** Zheyi Hu, Zaigang Zhou, Yahui Hu, Jinhui Wu, Yunman Li, Wenlong Huang

**Affiliations:** 1 State Key Laboratory of Natural Medicines, Department of Physiology, China Pharmaceutical University, Nanjing, 210009, China; 2 State Key Laboratory of Pharmaceutical Biotechnology, Nanjing University, Nanjing, 210093, China; 3 Center of Drug Discovery, China Pharmaceutical University, Nanjing, 210009, China; Columbia University, UNITED STATES

## Abstract

**Background:**

Multidrug efflux transporter P-glycoprotein (P-gp) is highly expressed on membrane of tumor cells and is implicated in resistance to tumor chemotherapy. HZ08 is synthesized and studied in order to find a novel P-gp inhibitor.

**Methods:**

MDCK-MDR1 monolayer transport, calcein-AM P-gp inhibition and P-gp ATPase assays were used to confirm the P-gp inhibition capability of HZ08. Furthermore, KB-WT and KB-VCR cells were used to evaluate the P-gp inhibitory activity of HZ08 both in vitro and in vivo.

**Results:**

Results showed that HZ08 was more potent than verapamil in MDCK-MDR1 monolayer transportation model. Meanwhile, P-gp ATPase assay and calcein-AM P-gp inhibition assay confirmed that HZ08 inhibited P-gp ATPase with a calcein-AM IC50 of 2.44±0.31μM. In addition, significantly greater in vitro multidrug resistance reversing effects were observed when vincristine or paclitaxel was used in combination with 10μM HZ08 compared with 10μM verapamil. Moreover, HZ08 could significantly enhance the sensitivity of vincristine with a similar effect like verapamil in both KB-WT and KB-VCR tumor xenograft models.

**Conclusions:**

The novel structure HZ08 could be a potent P-gp inhibitor.

## Introduction

The successful chemotherapy of solid and hematological tumors has been affected by intrinsic or acquired drug resistance, named as multi-drug resistance (MDR). Multidrug resistant tumors are found to be cross-resistant to a broad, but well-defined spectrum of structurally and functionally unrelated cytotoxic drugs, such as anthracyclines, epipodophyllotoxines, vinca alkaloids, colchicin, and taxanes.[1,2] In most cases, the cross resistance profile has been shown to be accompanied by a decrease in drug accumulation of the resistant cells, which is due to active efflux of these drugs by the multidrug transporter P-glycoprotein (P-gp).[3,4]

P-gp is a type of ATPase and an energy-dependent trans-membrane drug efflux pumpconsisted of 1480 amino acids. It is an important member of the ATP-binding cassette (ABC) transporters.[5,6] Several studies have demonstrated the possibility of using P-gp inhibitors to reverse the P-gp mediated efflux MDR in an attempt to improve the efficiency of chemotherapeutic agents as well as the pharmacokinetic and pharmacodynamic profiles of a number of challenging molecules, especially potent cancer curing compounds. This concept offers new opportunities to overcome drug-drug interactions exhibited by a combination of P-gp substrates/inhibitors, resulting in a refined drug absorption, distribution, metabolism and improved pharmacokinetics. Therefore, inhibiting the function of P-gp is thought to be one of the most useful method to reverse the acquired MDR.[7]

In general, the activity of P-gp can be inhibited either by blocking drug binding site competitively or by interfering ATP hydrolysis.[8,9] Most of the inhibitors, such as verapamil (VER), inhibit P-gp function by blocking drug binding sites. The mechanism for this kind of inhibitors is similar as P-gp handling its substrates, if these compounds are only mediated through binding sites. Moreover, a significantly higher dosage is usually needed to serve as a good P-gp inhibitor for these drugs, which can lead to unexpected side effects.[10,11] On the other hand, compounds inhibiting ATP hydrolysis may serve as better inhibitors since ATP binding and hydrolysis has been found to be essential for P-gp function, where one molecule of drug is effluxed at the expense of two molecules of ATP.[4] These drugs are unlikely to be transported by P-gp and a lower dose is required to achieve favorable P-gp inhibition, especially when used locally at gut lumen and cancer.[12]

**Fig 1:**
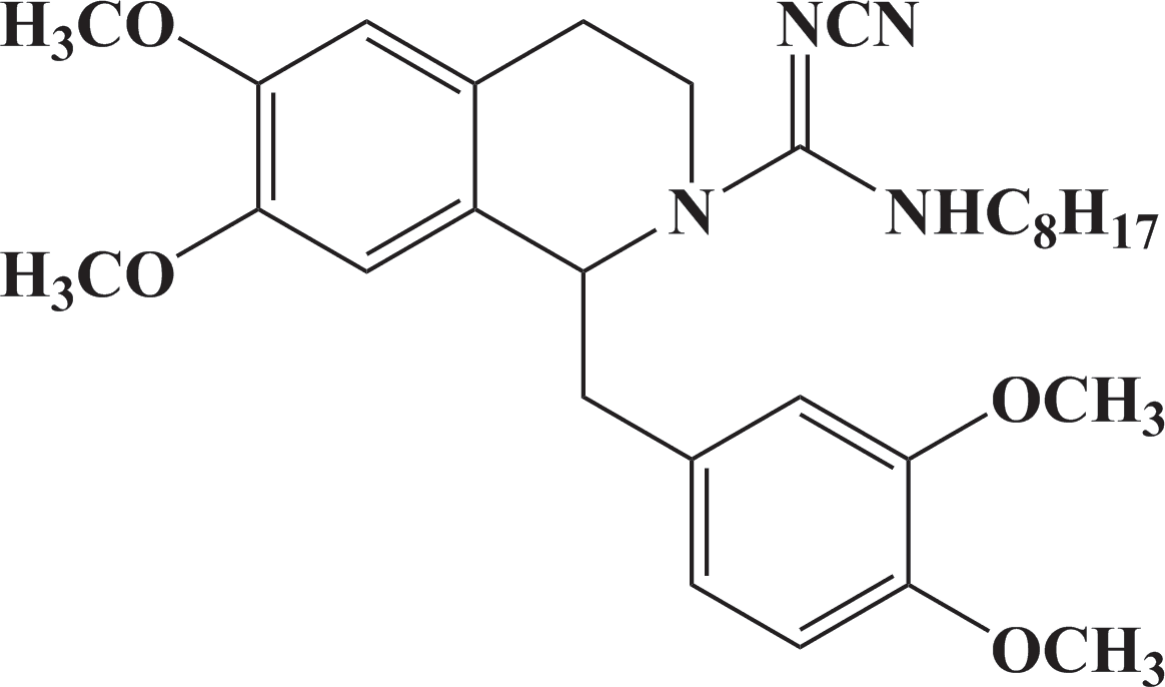
The chemical structures of HZ08.

HZ08 (Fig. 1) was designed and synthesized based on tetraisohydroquinoline as a new P-gp inhibitor and novel MDR modulator in order to reverse cancerous multidrug resistance.[13–15] Tetraisohydroquinoline and its derivatives have been demonstrated to have P-gp inhibition ability.[16,17] Investigations have indicated important structural features of molecules that modulate the function of ABCB1, namely two planar aromatic domains and a basic nitrogen atom within an extended aliphatic chain, a bulky aromatic ring system with a heteroatom in the third position toward the anthranilamide nucleus at the opposite end of the tetrahydroquinoline group, hydrophobicity, and nitrogen or hydrogen bond acceptor groups.[18–21] Meanwhile, tetraisohydroquinoline and its derivatives have been shown to be potent inhibitors of ATPase, which was important in the ABC transporters caused MDR.[22,23] Therefore, HZ08 may show high activity as P-gp inhibitor since it is an ideal tetraisohydroquinoline derivative with an extended aliphatic chain that well coincide the important structural features of molecules that modulate the function of P-gp.

Previous studies have reported the reversal effect of HZ08 on some multidrug resistant cell lines and speculated its mechanism probably related to cycle arrest, apoptosis sensitization or inhibits P-gp as its substrate. However, HZ08 were seldom investigated in animal models and the other possible mechanisms remain unclear.[24–26] In present studies, MDCK-MDR1 monolayer transportation model was used to evaluate the P-gp inhibit effect of HZ08, P-gp ATPase assay was performed to investigate the mechanism P-gp inhibition by HZ08, and the potency of HZ08 was characterized by calcein-AM P-gp inhibition assay.[27,28] Finally, in vitro cell inhibition and in vivo tumor growth inhibition assay were carried out to evaluate the P-gp inhibition of HZ08 co-treated with specific cancer chemotherapy drugs and the results were compared with VER.

## Materials and Methods

### Chemicals and materials

HZ08 was dissolved in dimethylsulfoxide (DMSO, Sigma-Aldrich) for in vitro assays. Doxorubicin (DOX) and VER were obtained from Becton Dickinson. GF120918, amprenavir (AMP), and paclitaxel (PTX) were purchased from Sigma-Aldrich. Vincristine (VCR) was purchased from J&K Scientific. Cell Counting Kit-8 (CCK-8) was purchased from Dojindo Molecular Technologies Inc. Pgp-Glo assay systems were purchased from Promega Corporation.

### Cell lines

KB-WT (human epidermoid carcinoma, wide-type), KB-VCR (resistant to VCR) and MDCK-MDR1 were purchased from the Type Culture Collection of the Chinese Academy of Sciences, Shanghai, China.

### MDCK-MDR1 monolayer transport assay

MDCK-MDR1 cells could differentiate into columnar epithelium with tight junctions in three days later to form cell monolayer. P-gp was significantly higher expressed on the apical side of the MDCK-MDR1 monolayer, while it was not expressed on the basal side. Thus, only the apical side of the membrane possessed P-gp medicated drug efflux capability.[29,30] If a drug was a P-gp substrate, its permeation from apical side (A) to basal side (B) would be inhibited and reduced, while the permeation from B to A would not be affected. When a P-gp inhibitor was added, the permeation of P-gp substrate from A to B could be increased. MDCK-MDR1 cell monolayer was hereby used to predict the P-gp inhibition ability of HZ08.

MDCK-MDR1 cells were cultured in Dulbecco's MEM (DMEM) Medium supplemented with 10% fetal bovine serum (FBS) and 2 mM L-glutamine. Cells were kept at 37°C in 5% CO_2_/95% air and split twice weekly at a ratio of 1:10 when they reached 80–90% confluence. For transport studies, cells were seeded onto Falcon 24-multiwell insert system at a density of 6x10^5^ cells/mL in 0.45mL and the basolateral compartment contained 1.3 mL cell culture medium. The plates were incubated at 37°C with 5% CO_2_/95% air for 4 days and subsequently used for assays. Before the experiment, cell culture medium were replaced with transport medium (DMEM containing no phenol red or sodium pyruvate was used because sodium pyruvate has an effect on both expression of P-gp and drug accumulation.[31,32]) consisting of Lucifer yellow (100 μM), test compounds (AMP 5μM or VCR 1μM or HZ08 10μM) and with or without HZ08 (10 μM). Two specific P-gp inhibitors, GF120918 (2 μM) and VER (10 μM), were used as controls. Plates were incubated for 90 min in 5% CO_2_/95% air, 37°C CO_2_ incubator with slowly shaking (60 rpm). Apical and basolateral compartments were sampled at the end of the transport experiment and the tested substance concentrations in both compartments were determined by LC-MS/MS. The LC system comprised an Agilent 1200 series liquid chromatography (Agilent Technologies Inc., USA). Mass spectrometric analysis was performed using an Agilent 6410B (triple-quadrupole) instrument with an ESI interface. Lucifer Yellow was used as an internal control to verify the integrity of cell monolayer during the test and fluorescence measurements were detected using an excitation of 430 nm and an emission of 538 nm. Cell monolayer with Lucifer yellow Papp > 30 nm/s were discarded.[33]

The apparent permeability (P_app_) and efflux ratio were calculated based on the equations reported previously.[33]

### Calcein-AM P-gp inhibition assay

The calcein-AM assay was optimized from previous studies.[28,29] MDCK-MDR1 cells were cultured as described above. Cells were seeded at 8,000 cells/well (100μl of culture medium) in 96-well flat clear bottom black polystyrene plates. The medium was changed 24h after seeding and the assay was performed 48 h later. On the day of study, the medium was removed and plates were washed three times with phosphate-buffered saline (PBS). Test compounds at various concentrations were added to plates in 50μl of transport buffer containing 0.5% DMSO as solvent. Plates were incubated at 37°C for 30 min. Calcein-AM was added in 50μl of PBS to yield a final concentration of 10μM. After 15min incubation, plates were washed three times with ice-cold PBS. Finally, 100μl PBS was added to each well and the plates were read by a SpectraMax M5 microplate reader at excitation and emission wavelengths of 490 and 515nm.

P-gp inhibition was calculated as described previously.[29] The curves of inhibition rates were fitted with GraphPad Prism 5 and the IC50 values were calculated. Each assay was conducted three times in quadruplicate.

### P-gp ATPase activity assay

The inhibitory effects of HZ08 on ATPase activity were examined against a VER-stimulated ABCB1 ATPase activity estimated by Pgp-Glo assay systems. Sodium orthovanadate (Na_3_VO_4_) was used as an ABCB1 ATPase inhibitor. HZ08 at various concentrations was incubated with 0.1mM VER, 5mM MgATP, and 25μg of recombinant human ABCB1 membranes at 37°C for 40 min. Luminescence was initiated by ATP detection buffer. The plate was incubated at room temperature for 20 min to develop luminescent signal, and was read by a luminescence detector (EnVision Multilabel Reader 2104, PerkinElmer Inc.). The changes in relative light units (RLU) were determined by comparing Na_3_VO_4_-treated samples with HZ08 and VER combination-treated samples, and the ATP consumed was measured through comparison with a standard curve.[34]

### Cell cytotoxicity assay

KB-WT and KB-VCR cell lines were used in this assay. Briefly, cells (2,000/well) were seeded in 96-well plate and cultured at 37°C in 5% CO_2_ for 24h. Then various concentrations of HZ08 or VER were added to the medium with full range concentration of different chemotherapeutics (VCR, PTX or DOX). After 72 h of treatment, 15μL Cell Counting Kit-8 solution was added to each well followed by another 2 h of incubation. The absorbance at 450nm and 600nm were then measured using a SpectraMax M5 microplate reader. The curves of growth inhibition rate were fitted with GraphPad Prism 5 and the IC50 values were calculated. Each assay was conducted three times in quadruplicate

### Animals and tumor xenograft experiments

All the animal experiments were approved by the Institutional Animal Care and Use Committee at China Pharmaceutical University (Permit Number: CPU1201P) and carried out in strict accordance with the recommendations in the Guide for the Care and Use of Laboratory Animals of AAALAC international. Animals were acclimated for 5 days prior to study initiation. During the study, animals were observed for any clinically relevant abnormalities. If any animal was moribund due to treatment associated toxicity or tumor over-growth (≥ 2000 mm^3^) before scheduled sacrifice, it would be euthanized. At the endpoint, the animals were euthanized by using CO_2_ and all efforts were made to minimize suffering.

2×10^6^ KB-WT or KB-VCR tumor cells (in 0.1 mL, 1:1 with Matrigel) were inoculated subcutaneously into the right flank of 5,6-week-old female Balb/c nude mice (Shanghai SINO-British SIPPR/BK Lab Animal Ltd, China). From 10 to 14 days after injection, animals were randomized and treated with weekly intravenous injection of VCR (2 mg/kg bodyweight), the combination of VCR plus VER (2 mg/kg bodyweight), the combination of VCR plus HZ08 (2 or 5 mg/kg bodyweight) or an equal volume of vehicle, respectively. Body weight and the tumor growth were monitored twice a week. Tumor size was measured to the nearest 0.1 mm using vernier caliper and applying the formula: Tumor volume = length × width^2^ × 0.5. Efficacy data was graphically represented as the mean tumor volume ± standard error of the mean (SEM). Tumors were collected for further analysis after last measurement.

## Results

### MDCK-MDR1 monolayer transport assay

In present study, AMP was firstly used as the standard P-gp substrate to evaluate whether HZ08 could inhibit its transport.[35–37] As shown in Table 1, the P_app(B→A)_ of 5μM AMP across MDCK-MDR1 cell monolayer was 479.7±45.7nm/s, which was higher than P_app(A→B)_ of 11.6±1.3nm/s, exhibiting a efflux ratio of 42.0±7.2. When a typical P-gp inhibitor, VER (10μM) or GF120918 (2μM) was added, the efflux ratio of AMP was decreased to 10.6±2.8 or 0.9±0.1, respectively. Theoretically, after adding a compound, if the efflux ratio of a P-gp substrate was significantly decreased but still significantly larger than 1, the compound was considered as a P-gp inhibitor. If the efflux ratio was decreased to nearly 1, the added compound was considered as a potent P-gp inhibitor.[28,29] Results from the present assays indicated that GF120918 at 2μM could be taken as a potent P-gp compared to VER at 10μM. In terms of HZ08, an obviously increased P_app(A→B)_ of 266.6±94.5nm/s was obtained. The efflux ratio decreased to 1.7±0.2, indicating that 10μM HZ08 was a potent P-gp inhibitor.

**Table 1:** Monolayer transport assay across the MDCK-MDR1 cells.

	P_app(A→B)_ (nm/s)	P_app(B→A)_ (nm/s)	Efflux Ratio
AMP 5μM	11.6±1.3	479.7±45.7	42.0±7.2
+10 μM VER	49.2±12.8**	496.0±11.4	10.6±2.8
+2 μM GF120918	359.2±105.6**	335.3±86.0	0.9±0.1
+10 μM HZ08	266.6±94.5**	427.9±81.8	1.7±0.2
VCR 1μM	3.2±1.0	19.9±2.8	6.4±1.1
+10 μM VER	8.7±2.9*	18.7±6.5	2.1±0.3
+2 μM GF120918	10.1±2.1**	11.8±1.8*	1.2±0.1
+10 μM HZ08	10.9±2.9*	15.3±4.6	1.5±0.7

Data are presented as mean±SD, n = 3, Significance levels

* P<0.05

** P<0.01

***P< 0.001.

Another well-known P-gp substrate, VCR, was also selected to further evaluate the P-gp inhibition of HZ08. The P_app(B→A)_ of 1μM VCR was 19.9±2.8nm/s, which was higher than P_app(A→B)_ of 3.2±1.0nm/s, resulting an efflux ratio of 6.4±1.1. The efflux ratio decreased significantly when co-treating with 10μM of VER or 2μM of GF120918. Similarly, after adding HZ08, the efflux ratio of VCR was also decreased, which was in line with the results from AMP.

### HZ08 inhibits the P-gp ATPase activity

P-gp ATPase was necessary for ATP hydrolysis and the energy gained from ATP hydrolysis was essential for the drug-efflux function of P-gp. The higher the P-gp ATPase activity was, the more energy could be provide by the hydrolysis of ATP.[5,38] P-gp inhibitors could inhibit the ATPase activity, resulting in a decreased ATP consumption and energy production.[5,39]

**Fig 2:**
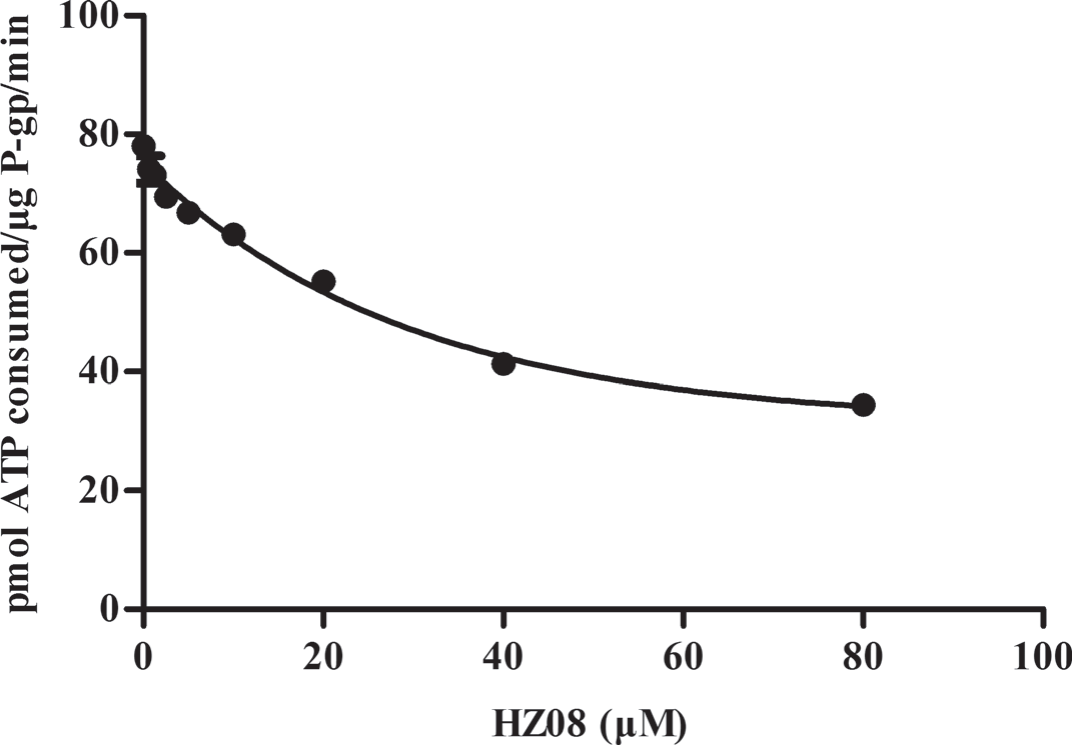
HZ08 inhibition of VER-stimulated P-gp ATPase activity. P-gp ATPase assays were performed according to the instruction of P-gp-Glo™ Assay Systems. Each point represents the mean±SD for triplated independent determinations.

In the present study, VER was used stimulate the ATPase activity. After the addition of VER, the ATP consumption was increased from 17.18±7.14 pmol/μg P-gp/min to 78.04±1.38 pmol/μg P-gp/min. However, the ATP consumption was significantly and dose-dependently decreased by HZ08 (Fig. 2 and Table 2). This reduction of VER-stimulated ATPase activity indicated that HZ08 was a P-gp ATPase inhibitor.

**Table 2:** HZ08 inhibition of VER-stimulated P-gp ATPase activity.

	ATP	Fold stimulation
	pmol ATP consumed/ μg P-gp / min	
Non-treated	17.18±7.14	/
VER(100μM)	78.04±1.38	4.54
+ HZ08 (0.625μM)	74.09±4.02	4.31
+ HZ08 (1.25μM)	73.10±1.34*	4.26
+ HZ08 (2.5μM)	69.42±1.31**	4.04
+ HZ08 (5μM)	66.82±0.87***	3.89
+ HZ08 (10μM)	63.11±1.71***	3.67
+ HZ08 (20μM)	55.21±0.43***	3.21
+ HZ08 (40μM)	41.25±3.00***	2.40
+ HZ08 (80μM)	34.48±1.56***	2.01

Data are presented as mean±SD, n = 3

*P<0.05

**P<0.01

***P< 0.001 compared to the value from VER single agent treatment wells.

### HZ08 inhibits calcein-AM efflux

Calcein-AM was a P-gp substrate and it could pass through the cell membrane.[40] Calcein-AM itself has no fluorescene. Once permeated into the cell, Calcein-AM would be hydrolyzed into fluorescent calcein by esterases. The produced calcein would be trapped in the cytoplasm of cells.[40] However, P-gp could efflux the calcein-AM out of the cells, resulting a decreased calcein production in cells and reduced fluorescence. When P-gp inhibitors were added, P-gp was inhibited, resulting an increase of calcein-AM and fluorescence in cells. [41–43] Therefore, the calcein-AM fluorescent assay can be used to evaluate the P-gp inhibition ability of potent P-gp inhibitors.[43]

**Fig 3:**
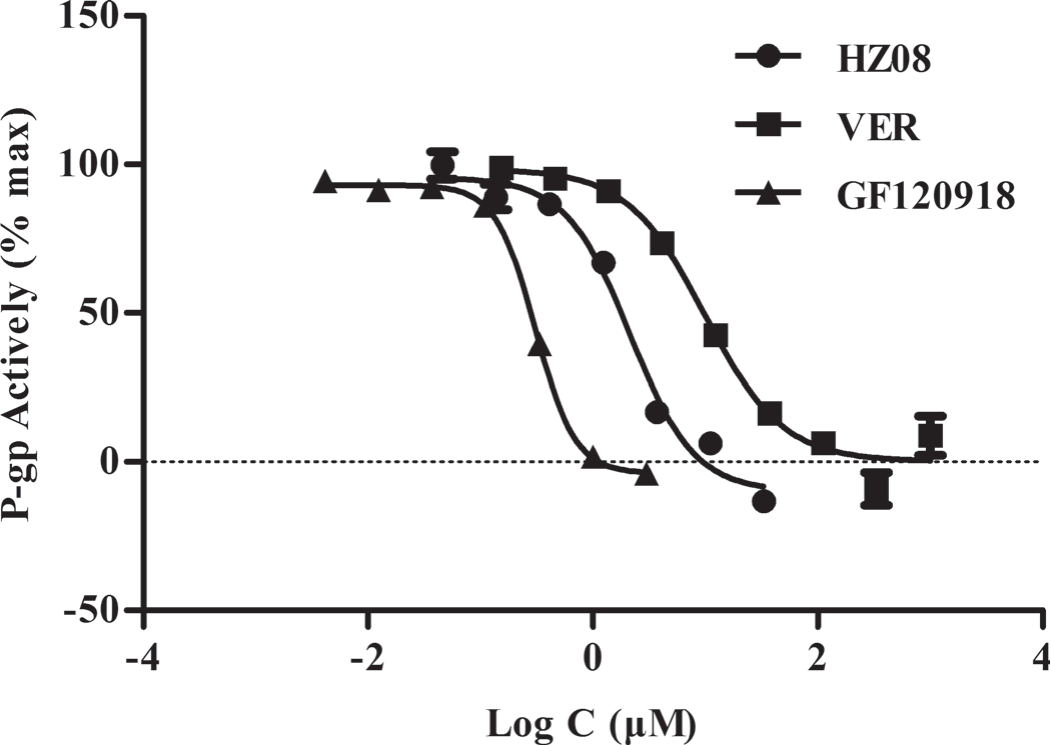
P-gp activity (% max) of VER, GF120918 and HZ08 detected by calcein-AM assay. Each point represents the mean±SD for triplated independent determinations.

GF120918 was known to completely inhibit P-gp at 2μM. At this concentration, the highest amount of calcein would be produced in cells and the max fluorescence intensity could be detected.[44,45] Therefore, the fluorescence intensity detected in 2μM GF120918 treated cells was considered as 100% in the present study and the P-gp activity was calculated based on the fluorescence intensity detected after adding different concentrations of P-gp inhibitors. Results showed that HZ08, GF120918 and VER decreased P-gp activity dose-dependently (Fig. 3). The IC50s of calcein-AM P-gp inhibition for HZ08, VER, and GF120918 were 2.44±0.31 μM, 9.52±0.33 μM, and 0.37±0.08 μM, respectively. The P-gp inhibitors were ranked as: potent inhibitors (IC50<10μM), moderate inhibitors (10μM ≤ IC50 < 50μM), and weak inhibitors (IC50≥50μM).[28,43] Therefore, HZ08 could be considered as a more potent P-gp inhibitor than VER (Table 3).

**Table 3:** calcein-AM P-gp inhibition IC50 (μM).

	IC50 (μM)
HZ08	2.44±0.31
VER	9.52±0.33
GF120918	0.37±0.08

Data are presented as mean±SD, n = 3.

### Reversal of P-gp mediated multidrug resistance in-vivo by HZ08

The cytotoxicity of HZ08 on KB-WT and KB-VCR cells was investigated by CCK-8 assay. When HZ08 was used as a single agent in CCK-8 assay, it showed no cytotoxicity to both cell lines at concentrations up to 20μM (data not shown).

The MDR reversing capability of HZ08 was then determined in KB-WT and KB-VCR cells. Three tumor chemotherapy drugs VCR, DOX and PTX were chosen as typical P-gp substrates. As shown in Table 4, VCR could inhibit the cell growth with a concentration dependent manner with an IC50 around 1.5nM. Only slight decreases in IC50 were observed after 10μM VER, 5μM HZ08 or 10 μM HZ08 were added, suggesting that the pumping out effects made by P-gp were not obvious in KB-WT cell lines.

**Table 4:** Effect of HZ08 on reversing KB-WT cell and ABCB1-medicated MDR KB-VCR cells.

Compound	KB-WT Cell		KB-VCR Cell		
	IC50 (μM)	FR	IC50 (μM)	FR
VCR	0.0015±0.0002	1.00	0.6187±0.1576	1.00
+10 μM VER	0.0008±0.0000**	1.83	0.0075±0.0015**	82.74
+10 μM HZ08	0.0004±0.0002**	3.67	0.0025±0.0010**	246.35
+5 μM HZ08	0.0009±0.0004	1.65	0.0036±0.0004**	173.46
+2.5 μM HZ08	0.0024±0.0021	0.64	0.0147±0.0097**	42.09
PTX	0.0037±0.0009	1.00	0.1935±0.0177	1.00
+10 μM VER	0.0018±0.0004*	2.03	0.0094±0.0010***	20.60
+10 μM HZ08	0.0014±0.0004*	2.68	0.0015±0.0000***	131.04
+5 μM HZ08	0.0019±0.0005*	1.97	0.0016±0.0007***	120.06
+2.5 μM HZ08	0.0020±0.0004*	1.85	0.0184±0.0085**	10.50
DOX	0.0473±0.0197	1.00	0.2447±0.0398	1.00
+10 μM VER	0.0372±0.0171	1.27	0.0763±0.0068**	3.21
+10 μM HZ08	0.0399±0.0166	1.19	0.0512±0.0103**	4.78
+5 μM HZ08	0.0573±0.0264	0.83	0.0637±0.0166**	3.84
+2.5 μM HZ08	0.0461±0.0251	1.03	0.0757±0.0278**	3.23

Data are presented by mean±SD of three independent experiments performed in quadruple. Fold-resistance (FR) was calculated by dividing IC50 value for cells with chemotherapeutic drugs without HZ08 or VER by that obtained in the presence of these two drugs.

* P<0.05

** P<0.01

***P< 0.001 compared to the value obtained without HZ08 or VER.

In KB/VCR cells, the IC50 of VCR was much higher than KB-WT cell line, corresponding with the fact that P-gp was extremely highly expressed on the membrane of KB-VCR cells. By co-treating with 10μM VER, 2.5μM HZ08, 5μM HZ08 or 10μM HZ08, the IC50 values were significantly decreased compared with VCR control group. These results indicated that VER and, HZ08 could significantly enhance the sensitivity of KB-VCR cells to cancer chemotherapy drug VCR. Meanwhile, the fold-resistance (FR) were 82.74 for 10μM VER and 243.65 for 10μM HZ08 suggesting that the MDR reversing capability of HZ08 was much higher than VER.

Another two typical P-gp substrates, DOX and PTX, were also selected to measure the multidrug resistance reversing ability of HZ08 in KB-WT and KB-VCR cells. HZ08 hardly changed the sensitization of cells to anticancer agents (DOX and PTX) in the KB-WT cells even at the maximum concentrations of 10μM, whereas it produced significant and dose-dependent decreases of IC50 of these drugs in KB-VCR cells (Table 4). In addition, the FR of 10μM HZ08 were 131.04 for PTX and 4.78 for DOX in KB-VCR cells, while the values were 20.6 for PTX and 3.21 for DOX when 10μM VER was used. These results also indicated that multidrug resistance reversing capability of HZ08 was much higher than VER at a concentration of 10μM.

Overall, HZ08 could better enhance the cytotoxicity of VCR, DOX and PTX to KB-VCR cells compared with VER, suggesting that HZ08 was a potent P-gp inhibitor.

### HZ08 reverses P-gp mediated multidrug resistance in vivo

In these studies, the antitumor enhancement by HZ08 was evaluated in Balb/c nude mice bearing KB-WT and KB-VCR xenografts.

**Fig 4:**
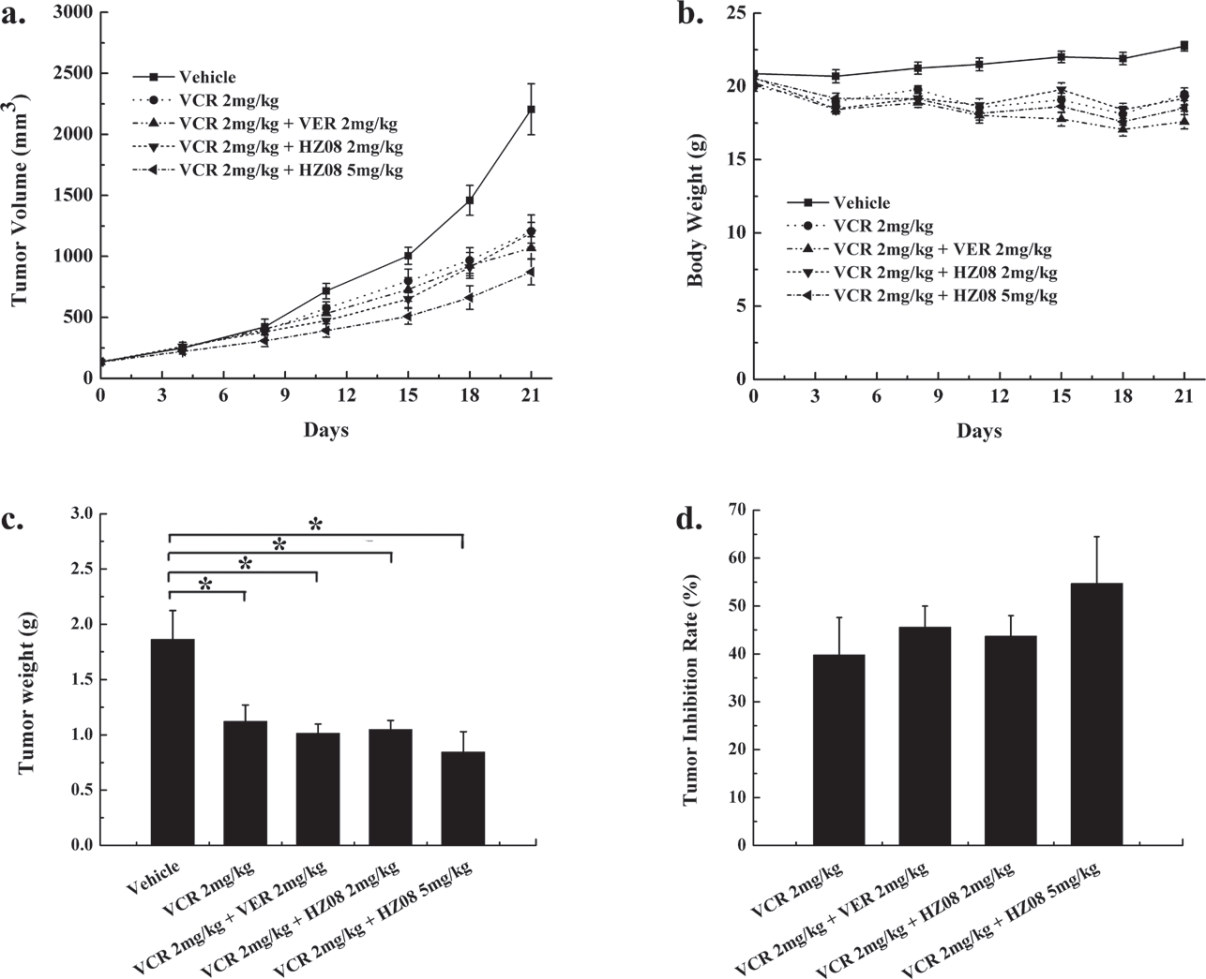
Effect of HZ08 in KB-WT xenograft tumor model in vivo. Mice were implanted subcutaneously (s.c.) with KB-WT cells and divided into five groups randomly: saline; 2mg/kg VCR; 2mg/kg VER plus 2mg/kg VCR; 2mg/kg HZ08 plus 2mg/kg VCR and 5mg/kg HZ08 plus 2mg/kg VCR. (a). Tumor volume; (b). Body weight; (c). Tumor weight of each mouse on day 21; (d) Tumor inhibition rate (%) on day 21. (mean±SEM, n = 8); *p<0.05, **p<0.01. Tumor inhibition rate (%) = 100 − (Tumor Weight_Treatment_/Tumor Weight_vehicle_) × 100.

In KB-WT xenograft model, the mean tumor volume in saline-treated control group reached 2205.64±209.34 mm^3^ (mean±SEM) on day 21 post treatment. On the other hand, the mean tumor volume of mice treated with VCR (2mg/kg) was significantly smaller compared to the vehicle group since day 18 (P<0.01) and it reached only 1206.41±132.53 mm^3^ (P<0.05) on day 21 (Fig. 4A). These results suggested that VCR had significant antitumor effects against KB-WT xenografts at 2 mg/kg. The growth of KB-WT xenografts were further delayed in animals treated with VCR in combination with VER or HZ08. The mean tumor volume for 2mg/kg VER or 2mg/kg HZ08 combined with VCR at day 21 were 1068.05±93.15 mm^3^ (P<0.05) and 1193.11±84.95 mm^3^ (P<0.05), respectively. No significant difference was seen among these two modulators. By adding 5mg/kg HZ08, a higher tumor inhibition was observed. The mean tumor volume for 5mg/kg HZ08 combined with VCR at day 21 were 872.92±301.72 mm^3^ (P<0.001). To further confirm the results, mice were euthanized by CO_2_ inhalation and tumors were dissected and weighted on day 21. (Fig. 4C) The highest tumor weight was seen in the vehicle group (1.866±0.259 g, mean±SEM) while it was 1.123±0.147 g for the VCR single treatment group. After adding VER (2mg/kg) or HZ08 (2 or 5 mg/kg), tumor weights were also significantly decreased compared with vehicle group (1.015±0.082 g, 1.050±0.080 g and 0.845±0.183 g, respectively). In this KB-WT model, there were no significant differences of tumor volume or tumor weight among VCR single treatment group, VER plus VCR combination group or HZ08 plus VCR combination groups corresponding with the fact that P-gp was lowly expressed in KB-WT cell. (Fig. 4D)

**Fig 5:**
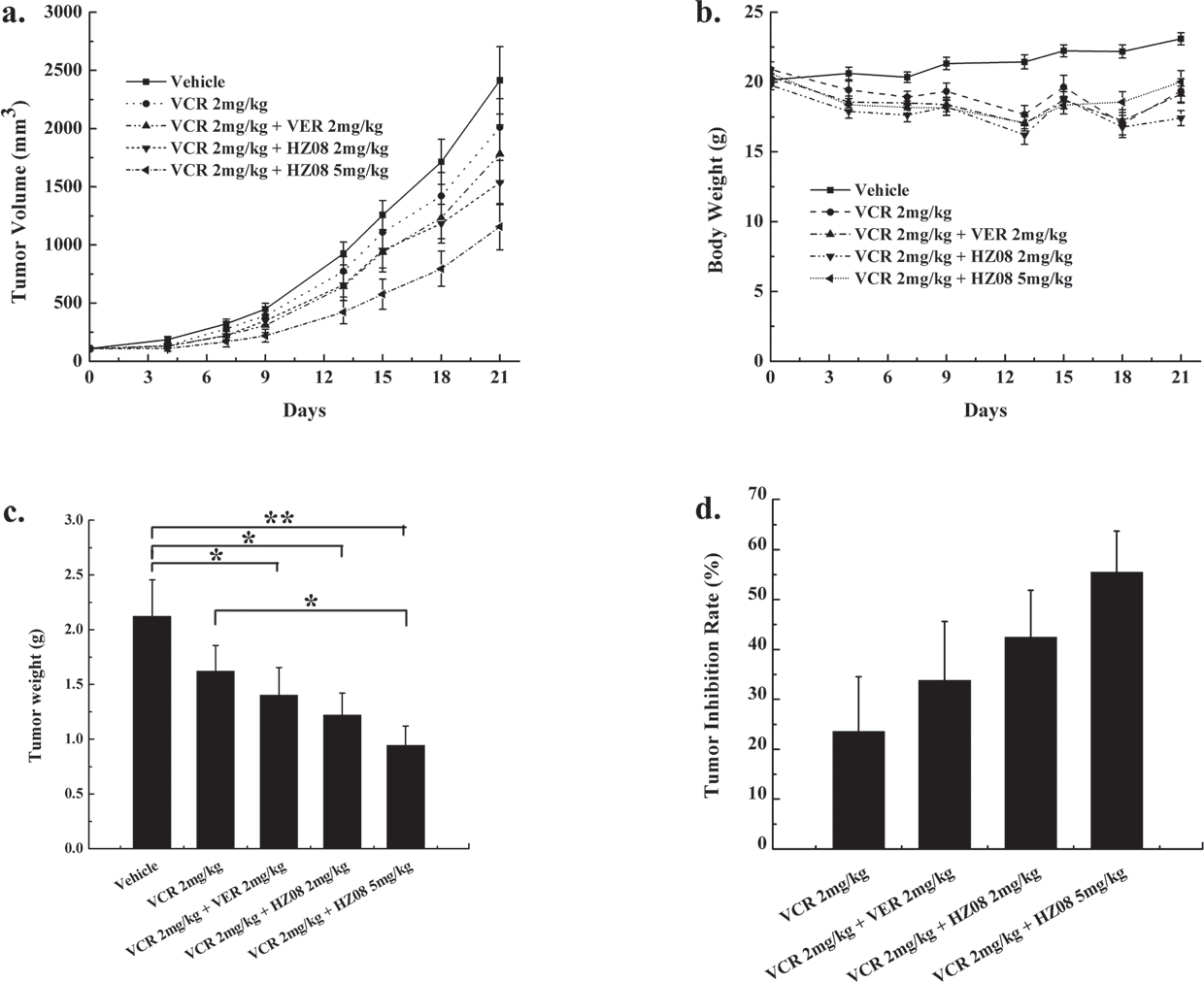
Effect of HZ08 in KB-VCR xenograft tumor model in vivo. Mice were implanted subcutaneously (s.c.) with KB-VCR cells and divided into five groups randomly: saline; 2mg/kg VCR; 2mg/kg VER plus 2mg/kg VCR; 2mg/kg HZ08 plus 2mg/kg VCR and 5mg/kg HZ08 plus 2mg/kg VCR. (a). Tumor volume; (b). Body weight; (c). Tumor weight of each mouse on day 21; (d) Tumor inhibition rate (%) on day 21. (mean±SEM, n = 8); *p<0.05, **p<0.01. Tumor inhibition rate (%) = 100 − (Tumor Weight_Treatment_/Tumor Weight_vehicle_) × 100.

In KB-VCR xenograft model, the mean tumor volume in vehicle group reached 2414.28±289.08 mm^3^ on day 21 post treatment. When mice were treated with 2mg/kg VCR, a little decrease in tumor volume could be observed and it reached 2009.99±24.57 mm^3^ (P>0.05) at day 21 (Fig. 5A). This indicated that VCR showed no significant antitumor effect at 2 mg/kg in KB-VCR tumor xenograft model. Moreover, when tumor bearing mice were treated with VCR plus 2 mg/kg VER, antitumor effect was slight increased (1783.90±248.00 mm^3^ at day 21) but still not reached the statistical significance compared with vehicle group. However, VCR combined with 2 mg/kg HZ08 could significantly increase the antitumor effect and at day 21 the tumor volume was 1536.36±190.04 mm^3^ (P<0.05). By increasing HZ08 dose to 5 mg/kg, a higher tumor inhibition was observed. Similarly, Mice were also sacrificed and tumors were dissected and weighted on day 21 (Fig. 5C). The highest tumor weight was observed in the saline group (2.124±0.330 g), while it was 1.623±0.233 g for the VCR group (P>0.05). The decreasing in tumor weight was limited when adding VER (1.405±0.250 g, P>0.05). On the other hand, when adding 2mg/kg or 5mg/kg HZ08, mean tumor weight was significantly decreased (1.222±0.200 g, 0.954±0.175 g, on day 21, respectively) compared with vehicle control group. In this KB-VCR model, HZ08 plus VCR showed a similar but more powerful tumor inhibition compared with VER plus VCR. (Fig. 5D).

Hence, the above two tumor xenograft models showed that HZ08 could enhance the chemotherapy sensitivity of tumor cells and its effect was similar to VER.

## Discussion

In the past several years, chemotherapy has become the most important and useful way to cure malignant cancer. But the development of MDR after a period of chemotherapy often made them useless.[46] Over-expression of ABC transporters has been shown to be responsible for MDR.[47] The most typical ABC transporters in the cell membrane was P-gp.[48,49] P-gp was able to limit the effectiveness of chemotherapy in a variety of common malignancies and was responsible for overall poor efficacy of cancer chemotherapy.[50,51] Therefore, reversing of multidrug resistance is thought to be a useful way to enhance the effects of chemotherapy.

In our investigation, MDCK-MDR1 cell monolayer assay was conducted. This assay could provide a measure of the specific human P-gp-mediated efflux activity by comparison of the efflux ratios of MDCK-MDR1 cell monolayer in the presence and absence of possible P-gp inhibitors.[43] In our experiment, HZ08 could significantly increase the permeation of AMP and VCR from apical side and basal side and the efflux ratios were decrease to nearly one. Meanwhile, a better P-gp inhibition efficacy was obtained by HZ08 compared with VER. Consequently, HZ08 is a potent P-gp inhibitor and its inhibition is better than VER.

P-gp could be inhibited mainly by blocking drug binding site or by interfering ATP hydrolysis.[8,9] Most of the known P-gp inhibitors, such as VER, inhibit its function by blocking drug binding sites. But this mechanism usually required, a significantly higher dosage in order to achieve effective P-gp inhibition, which usually led to unexpected side effect.[10,11] Some other P-gp inhibitors could inhibit the P-gp function through inhibiting ATPase activity as ATP hydrolysis was found to be essential for P-gp function.[4] These P-gp inhibitors were unlikely to be transported by P-gp and a lower dose was needed for a favorable P-gp inhibition especially when used locally at gut lumen and cancer.[12] To predict the possible mechanisms of HZ08 in P-gp inhibition, P-gp ATPase assay was conducted. The results showed that, HZ08 dose-dependently decreased VER-stimulated ATPase activity of P-gp (Fig. 2 and Table 2). HZ08 may thus inhibit the function of P-gp through inhibiting the ATP hydrolysis other than competing for the binding side. But, further studies are needed in order to unravel the exact mechanisms of HZ08 in P-gp inhibition.

Further, in vitro calcein-AM P-gp inhibiting assay was conducted. The in vitro calcein-AM P-gp inhibition assay can be used to detect compounds that inhibit P-gp-mediated efflux. In our experiment, the IC50 of calcein-AM P-gp inhibition for HZ08 was about 2.44±0.31 μM, while 9.52±0.33 μM for VER, suggesting that HZ08 is a potent P-gp inhibitor with a significantly better efficacy than VER (Table 3).

We determined the MDR reversing of HZ08 in KB-WT and KB-VCR cell lines. It was found that HZ08 markedly increased the sensitivity of KB-VCR cells to DOX, PTX, and VCR, but did not have these effects on KB-WT cells (Table 4). On KB-VCR cells, 10 μM HZ08 only little enhanced the sensitivity to DOX with a fold-resistance of 4.78, while it is 132 for PTX. PTX is a specific substrate of P-gp inhibitor, while DOX is a substrate of P-gp and MRP1.[47,52] The efflux of PTX is mainly mediated by P-gp, while the efflux of DOX is mediated by both P-gp and MRP1. Since both P-gp and MRP1 are highly expressed on KB-VCR cells, the limited enhanced sensitivity of KB-VCR cells to DOX may due to the specific inhibition of HZ08 to P-gp. Subsequent KB-WT and KB-VCR xenograft studies showed that HZ08 has similar antitumor enhancement ability in vivo compared with VER.

## Conclusion

In conclusion, our results indicate that the newly designed chemical structure HZ08 could enhance the permeation of VCR and AMP from AL to BL in MDCK-MDR1 cell monolayer. Meanwhile, P-gp ATPase assay and calcein-AM P-gp inhibition assay indicated that HZ08 is a potent P-gp inhibitor by inhibiting P-gp ATPase activity. Meanwhile, HZ08 could enhance the cytotoxicity and antitumor efficacy of VCR, DOX and PTX in vitro better than VER. Moreover, an equal enhancement of the cytotoxicity and antitumor efficacy of VCR was observed between HZ08 and VER. Hence, HZ08 might be a potent P-gp inhibitor.
